# Foot-and-mouth disease virus VP1 promotes viral replication by regulating the expression of chemokines and GBP1

**DOI:** 10.3389/fvets.2022.937409

**Published:** 2022-07-22

**Authors:** Li Yang, Hong Chen, Liqing Liu, Jingjing Song, Tian Feng, Yihan Li, Chao Shen, Lingbao Kong, Xiu Xin

**Affiliations:** ^1^Nanchang City Key Laboratory of Animal Virus and Genetic Engineering, Nanchang, China; ^2^Institute of Pathogenic Microorganism, Jiangxi Agricultural University, Nanchang, China; ^3^College of Bioscience and Engineering, Jiangxi Agricultural University, Nanchang, China; ^4^State Key Laboratory of Virology, College of Life Sciences, Wuhan University, Wuhan, China

**Keywords:** RNA-seq, transcriptome, gene expression, FMDV VP1, cytokine, GBP1

## Abstract

Foot-and-mouth disease virus (FMDV) is an acute, highly contagious, and economically destructive pathogen of vesicular disease that affects domestic and wild cloven-hoofed animals. The FMDV VP1 protein is an important part of the nucleocapsid and plays a significant role during FMDV infection. However, the signal pathways mediated by VP1 in the life cycle of FMDV and the related mechanisms are not yet fully understood. Here, we performed RNA-seq to compare gene expression profiles between pCAGGS-HA-VP1 transfected PK-15 cells and pCAGGS-HA (empty vector) transfected PK-15 cells. The results showed 5,571 genes with significantly different expression levels, of which 2,981 were up-regulated and 2,590 were down-regulated. GO enrichment analysis showed that 51 GO terms were significantly enriched in cell components including protein complex, membrane and organelle part. KEGG enrichment analysis showed 11 KEGG pathways were significantly enriched which were mainly related to the immune system, infectious viral disease, and signal transduction. Among the up-regulated genes, the chemokines such as CCL5, CXCL8, and CXCL10 in turn promoted FMDV replication. In contrast, GBP1, an interferon-stimulated gene that was suppressed by VP1 and FMDV, could effectively inhibit FMDV replication. Our research provides a comprehensive overview of the response of host cells to VP1 protein and a basis for further research to understand the roles of VP1 in FMDV infection including the genes involved in FMDV replication.

## Introduction

Highly contagious pathogens are a global biosecurity threat due to their high morbidity and mortality rates and their ability to rapidly spread epidemics that are difficult to control (1). Highly contagious Foot-and-mouse disease virus (FMDV) is a representative of such kind of pathogens with a broad host spectrum, ranging from ungulate species to other livestock, which has caused enormous economic losses (2).

As a member of the genus *Aphthovirus* of the family *Picornaviridae*, FMDV is a single-stranded positive-sense RNA virus with a genome of 8.5 kb (3). FMDV display diversity in genetic and phenotypic traits. In terms of the serotype, FMDV can be divided into seven groups: A, O, C, Asia1, South African Territories 1 (SAT1), SAT2, and SAT3 with a large number of subtypes. FMDV genome encodes four structural proteins (VP1, VP2, VP3, and VP4) and at least 10 non-structural proteins (L, 2A, 2B, 2C, 3A, 3B1, 3B2, 3B3, 3C, 3D, and some intermediate precursors) (4). As virion structure components, VP1-VP3 compose the outer capsid, while VP4 is located inside the capsid (5).

FMDV VP1 protein, a momentous component of the nucleocapsid, is essential for viral attachment and entry into host cells (6, 7). VP1 protein also serves as the dominant immunogenic component, which has maximum accessibility on the capsid surface. It contains at least two key epitopes located in the G-H loop of amino acids 141–160 and at the C-terminal site of amino acids 200–213 (8). The G-H loop contains an arginine-glycine-aspartic acid (RGD) triplet structure, mediating the engagement of the virus to the host cells through the integrin receptors (αvβ1, αvβ3, αvβ6, and αvβ8) (9, 10). Due to its importance in virus adsorption and entry, protective immunity, and serum specificity, VP1 has been used to define the genetic characteristics of the FMDV strain (11). A considerable amount of studies have proved that single amino acid replacements in FMDV VP1, especially near the RGD motif or the five-fold symmetry axis of the icosahedral virion, may contribute to the receptor recognition (12) and acid-induced virus uncoating (13–15), virus replication and pathogenicity (16, 17).

In addition to mediating receptor binding, VP1 also affects the signal pathways in the host cells, thereby facilitating viral replication. Studies have found that VP1 protein promotes host cells apoptosis by inhibiting the activation of the AKT pathway (18). It inhibits the production of IFN-I in cells by binding to sorcin protein and TPL2 protein (19–21). Besides, VP1 protein also competitively binds to MAVS with TARF3 to suppress the innate immune response, but the VP1 (E83K) mutation appears losing of this competitive inhibitory ability during the culture process (22). Recent studies have shown that the interaction between VP1 and the host ribosomal protein SA activates MAPK signaling pathway, thus facilitating FMDV replication (23). However, the comprehensive and systematic influence on the signal pathways in host cells regulated by the FMDV VP1 remains unclear.

To gain insight into the potential VP1-mediated signal pathways and how these pathways affect FMDV replication, we adopted RNA-seq to analyze the changes in the host gene transcriptional profile in transfected VP1-expressing PK-15 cells for the first time. Results of this study shed a light on FMDV pathogenesis.

## Materials and methods

### Virus and cells

The O-type FMDV (Akesu/58/2002, GenBank Accession No. AF511039) in the laboratory was provided by the Lanzhou Veterinary Research Institute of the Chinese Academy of Agricultural Sciences (24). PK-15 cells (CCTCC) were cultured in Dulbecco's Modified Eagle's Medium (DMEM) (Solarbio, China) supplemented with 10% fetal bovine serum (FBS) (EveryGreen, China) at 37°C with 5% CO_2_.

### Plasmid construction and transfection

Total RNA of FMDV-infected PK-15 cells was extracted with the TRIZOL reagent (TransGen Biotech, Beijing), then reverse transcribed into cDNA with oligo dT primers and M-MLV enzyme (TaKaRa, Japan) according to the manufacturer's instructions. VP1 and GBP1 genes were amplified with specific primers (Table 1) and LA-Taq^®^ polymerase (TaKaRa, Japan) by PCR. The PCR reaction was run for 35 cycles of 94°C for 30 s, 54°C for 30 s, and 72°C for 1min. PCR products were purified and digested by restriction endonuclease, and then ligated into restriction endonuclease digested pCAGGS-HA vector. The resulting plasmid was named pCAGGS-HA-VP1 or pCAGGS-HA-GBP1. The constructed plasmids were verified by DNA sequencing. The pCAGGS-HA-CCL5, pCAGGS-HA-CXCL8, and pCAGGS-HA-CXCL10 plasmids were constructed and preserved by our laboratory (25).

**Table 1:** Primers for plasmid construction and real-time PCR.

**Gene name**	**Primer**	**Primer sequence (5′ → 3′)**	**Usage**
VP1	F	CGGAATTCATGACCACCTCCCCGGGCGAGTCTG (*EcoRI*)	pCAGGS-HA-VP1
VP1	R	CCCTCGAGTTACTGTTTTGCGGGTGCCACAATC (*XhoI*)	pCAGGS-HA-VP1
GBP1	F	GCTCGAGATGGCCTCAAAGGTGCAC (*XhoI*)	pCAGGS-HA-GBP1
GBP1	R	GAAGATCTTTAGCTCAGGAAACATTCTTTCTTTG (*BglII*)	pCAGGS-HA-GBP1
RSAD2	F	CCCAGTGTCAGCATCGTGAG	qRT-PCR
RSAD2	R	ATAATCCCTGCACCACGTCC	qRT-PCR
CCL5	F	TCCCCATATGCCTCGGACAC	qRT-PCR
CCL5	R	CACACACCTGGCGGTTCTT	qRT-PCR
CXCL8	F	CTTCCAAACTGGCTGTTGCC	qRT-PCR
CXCL8	R	TGGAAAGGTGTGGAATGCGT	qRT-PCR
CCL4	F	ATGAAGCTCTGCGTGACTGT	qRT-PCR
CCL4	R	TTCCGCACGGTGTATGTGAA	qRT-PCR
CCL2	F	GTGTCCTAAAGAAGCAGTGATCTTC	qRT-PCR
CCL2	R	TCTGAGGGTATTTAGGGCAAGT	qRT-PCR
CXCL10	F	ATAAGGATGGGCCGGAGAGA	qRT-PCR
CXCL10	R	GTGGGAGCAGCTAACTTGGT	qRT-PCR
TP53	F	GGGTAACCTTGCGGTGTGAT	qRT-PCR
TP53	R	AGGGAGACTGCCCCTTCTTA	qRT-PCR
GAPDH	F	AAGCATGTGGGGGACTTGGA	qRT-PCR
GAPDH	R	AGTTAAAAGCAGCCCTGGTGA	qRT-PCR
TBK1	F	CCTTCGTCCAGTGGATGTTCA	qRT-PCR
TBK1	R	CTCCCACATGGACAAAATTCCA	qRT-PCR
GBP1	F	CTCACCCCAAGAAGCGAGAA	qRT-PCR
GBP1	R	CTGGTTAATGGTCCCCATGCT	qRT-PCR
SAT1	F	TTCGGAGAGCACCCCTTCTA	qRT-PCR
SAT1	R	GTTTGCCAATCCATGGGTCAT	qRT-PCR
EREG	F	GGTGTCAGATGCGAGCACTT	qRT-PCR
EREG	R	GACCCCTTGAGGACACTCTT	qRT-PCR
PLAT	F	ACAATGCAAACCTGCACGAC	qRT-PCR
PLAT	R	GTCACGGTCTCGTGTTGTCT	qRT-PCR
SERPINE1	F	ATATGACCAGACTCACCCGC	qRT-PCR
SERPINE1	R	AGACTTGAGAAGTCCGCCTG	qRT-PCR
SLC5A3	F	CGCTACGAGCTGGCTTTGAT	qRT-PCR
SLC5A3	R	CGCTACGAGCTGGCTTTGAT	qRT-PCR
AREG	F	CGTGGTGCTGTCACTCTTGA	qRT-PCR
AREG	R	CATTTCGCTAGCAGGGGGAG	qRT-PCR
GSK3A	F	AATGGCTCATTTGGGGTCGT	qRT-PCR
GSK3A	R	TTGCAGTGGTCCAGCTTACG	qRT-PCR
GSK3B	F	CGAGACACACCTGCACTCTT	qRT-PCR
GSK3B	R	CCGGCATTAGTATCTGAGGCT	qRT-PCR

PK-15 cells were transfected with the pCAGGS-HA-VP1 or empty plasmid for 36 h. The protein expression level of VP1 was detected and verified by western blot. Then the lysis using the TRIZOL reagent of these cells was sent to Beijing Novogene Illumina Biological Information Technology Co. Ltd. for RNA-Seq.

The overexpression levels of plasmids were evaluated by qRT-PCR or western blot after 24 h with the pCAGGS-HA-CCL5/CXCL8/CXCL10/GBP1 plasmid transfection into PK-15 cells. Then the transfected cells were infected with FMDV at the multiplicity of infection (MOI) of 0.1. Cells were harvested at different times post-infection, and the expression of FMDV 3D was measured by qRT-PCR or western blot.

### Virus infection

The PK-15 monolayer cells were incubated with FMDV at an MOI of 0.1 or the same volume of DMEM (FBS-free) as control at 37°C. The supernatant was removed and added 2% fetal bovine serum culture medium 1 h later. Then 250 ul TRIZOL Reagent (TransGen Biotech, Beijing) was added to each dish and cells were collected at 4, 8, and 12 h post-infection, and total RNA was extracted according to the manufacturer's protocol for subsequent qRT-PCR analysis.

### Western blot analyses

After harvesting cells using RIPA lysis buffer, the samples were heated at 100°C for 15 min. Protein samples were separated by sodium dodecyl sulfate-polyacrylamide gel electrophoresis and transferred to PVDF membranes under constant flow at low temperatures. Blocking was performed with 5% skimmed milk for 1 h at room temperature. After overnight incubation at 4°C with the corresponding primary antibodies [anti-HA: 1:5,000, Proteintech; anti-3D (rabbit polyclonal antibody provided by the CCTCC) (26)], they were incubated at room temperature with the corresponding secondary antibodies [Horseradish peroxidase (HRP)-coupled anti-mouse or anti-rabbit secondary antibodies: 1:5,000, Proteintech]. Antigen-antibody complexes were detected using the Chemic DOC imaging system (BioRad, USA).

### Library construction and sequencing

The library started with RNA as total RNA, enriched the mRNA with polyA tail through Oligo (dT) magnetic beads, and then randomly interrupted the resulting mRNA with two-valent cations in NEB Fragmentation, according to the NEB standard library construction method (27). After the library was built, the library was initially quantified using Qubit2.0 Fluorometer, diluted to 1.5 ng/ul, and then the library's insert size was detected using Agilent 2100 bioanalyzer, and qRT-PCR accurately quantified the library's effective concentration (the library's effective concentration was higher than 2 nM) to ensure library quality. After the library inspection passed, the different libraries were pooled according to the effective concentration and the demand of the target down-machine data volume, and the end reading of 150 bp pairing was generated bioinformatic analysis. Data of the transcriptome profiles have been submitted to NCBI (Accession Number PRJNA833896).

### Sequencing information analysis

To ensure the quality and reliability of data analysis, clean data (clean reads) were obtained by removing reads containing adapter, reads containing ploy-N and low quality reads from raw data. Then the effective base (clean bases) was calculated for Q20, Q30, and GC content. The inter-sample correlation coefficient (Pearson correlation coefficient) was tested in R language to ensure repeatability between samples. For samples with biological repetition, the differential expression analysis between the two comparison combinations was performed using DESeq2 software. A *q*-value < 0.05 and |log2(foldchange)| > 1 was set as the threshold for significantly different expression. The expression patterns of the selected candidate genes were then observed by stratification analysis using Cluster 3.0 software. Through the cluster profile (3.4.4) software, the difference gene set was analyzed by padj < 0.05 as the threshold of significant enrichment. The differentially Expressed Genes (DEGs) were then subjected to Gene Ontology (GO) functional enrichment analysis and Kyoto Encyclopedia of Genes and Genome (KEGG) pathway analysis (28).

### qRT-PCR validation of DEGs

Reverse transcription reaction of the same RNA templates was performed using the PrimeScript TM RT Master Mix (Takara Biotechnology, China). RT-qPCR was performed using a gene-specific primer pair (Table 1) and TB Green^®^Premix Ex Taq™II (Takara Biotechnology, China) on an Applied Biosystems^®^ 7500 Fast Real-time System. The expression level of each gene relative to the reference gene GAPDH was calculated by the 2^−Δ*ΔCt*^ algorithm.

### Statistical analysis

Experimental data are presented as mean ± SE. Statistical significance between two groups was determined by equal variance two-tailed Student's *t*-tests. *P* < 0.05 was considered statistically significant. *P* < 0.01 was considered extremely significant.

## Results

### Construction recombinant plasmid expressing VP1 in PK-15 cells

**Figure 1:**
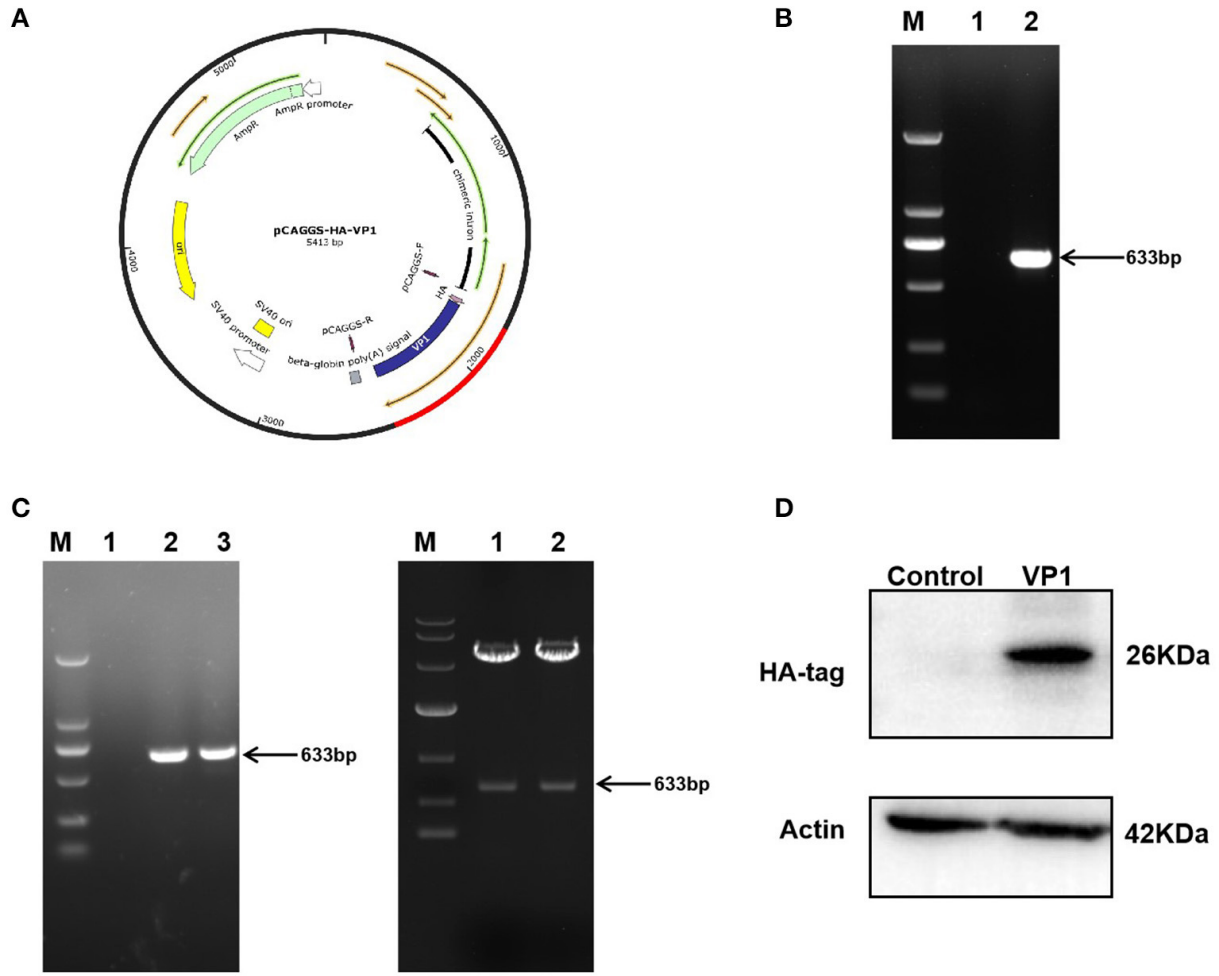
Construction and expression of pCAGGS-HA-VP1 Plasmid. **(A)** Model of a cloned fragment. **(B)** Target fragment VP1 amplified by PCR (lane 2), respectively. **(C)** The product of recombinant plasmid pCAGGS-HA-VP1 verified by colony PCR (lanes 2 and 3 on the left panel) and digested with EcoR1 and Xho1 (lanes 1 and 2 on the right panel) was observed with agarose gel electrophoresis. **(D)** The pCAGGS-HA vectors (control) or pCAGGS-HA-VP1 plasmids were transfected in PK-15 cells for 36 h, respectively, and detected by western blot analysis.

The pCAGGS-HA-VP1 plasmid was constructed using gene cloning (Figure 1A). The target fragment 633 bp was verified by agarose gel electrophoresis, and the plasmid was correct by DNA sequencing (Figures 1B,C). VP1 protein expression in PK-15 cells transfected with the plasmid for 36 h was detected by western blot using HA antibody. The result indicated that VP1 was highly expressed at 36 h post-transfection (Figure 1D). In summary, the pCAGGS-HA-VP1 plasmid was successfully constructed and expressed in PK-15 cells.

### Quality control of sequencing data

RNA from pCAGGS-HA-VP1 transfected PK-15 cells and pCAGGS-HA (empty vector) transfected PK-15 cells (three replicates for each group) were extracted. RNA integrity was assessed using the RNA Nano 6000 Assay Kit of the Bioanalyzer 2100 system. After cluster generation, the library preparations were sequenced on an Illumina Novaseq platform. The average raw reads for the VP1 group samples was 42,524,827 and the average raw reads for the control group samples was 44,359,931. We obtained an average of 41,388,887 clean reads from the VP1 group, and 42,957,732 clean reads from the control group after filtering out the low-quality reads. The percentage value of Q20 and Q30 was higher than 97.72 and 93.98%, respectively (Table 2), fulfilling the experimental requirements. The percentage of clean reads aligned to the reference genome was higher than 94.74% (Table 3). Among them, the percentage of uniquely mapped reads and multiple mapped reads were up to 90.09 and 3.88%, respectively. These data suggested a high quality of sequencing, which was reproducible and available for subsequent analysis.

**Table 2:** Statistical analysis of sequencing data.

**Sample**	**Raw data**	**Clean reads**	**Clean bases**	**Error rate**	**Q20/%**	**Q30/%**	**GC content/%**
Control-1	45,438,984	43,946,096	6.59G	0.03	97.81	94.23	53.32
Control-2	41,621,344	40,397,528	6.06G	0.02	97.94	94.47	53.05
Control-3	46,019,466	44,529,572	6.68G	0.03	97.72	93.98	53.22
VP1-1	45,801,536	44,584,606	6.69G	0.02	97.93	94.5	53.81
VP1-2	41,178,510	40,058,162	6.01G	0.03	97.86	94.34	53.96
VP1-3	40,594,434	39,523,892	5.93G	0.02	97.97	94.56	54.17

**Table 3:** Alignment results.

**Sample**	**Total reads**	**Total map reads %**	**Unique map reads %**	**Multimap reads %**
Control-1	43,946,096	95.31	91.15	4.16
Control-2	40,397,528	95.34	91.23	4.11
Control-3	44,529,572	95.13	90.09	4.23
VP1-1	44,584,606	94.91	91.02	3.89
VP1-2	40,058,162	94.74	90.86	3.88
VP1-3	39,523,892	95.11	91.21	3.91

### Analysis of DEGs

**Figure 2:**
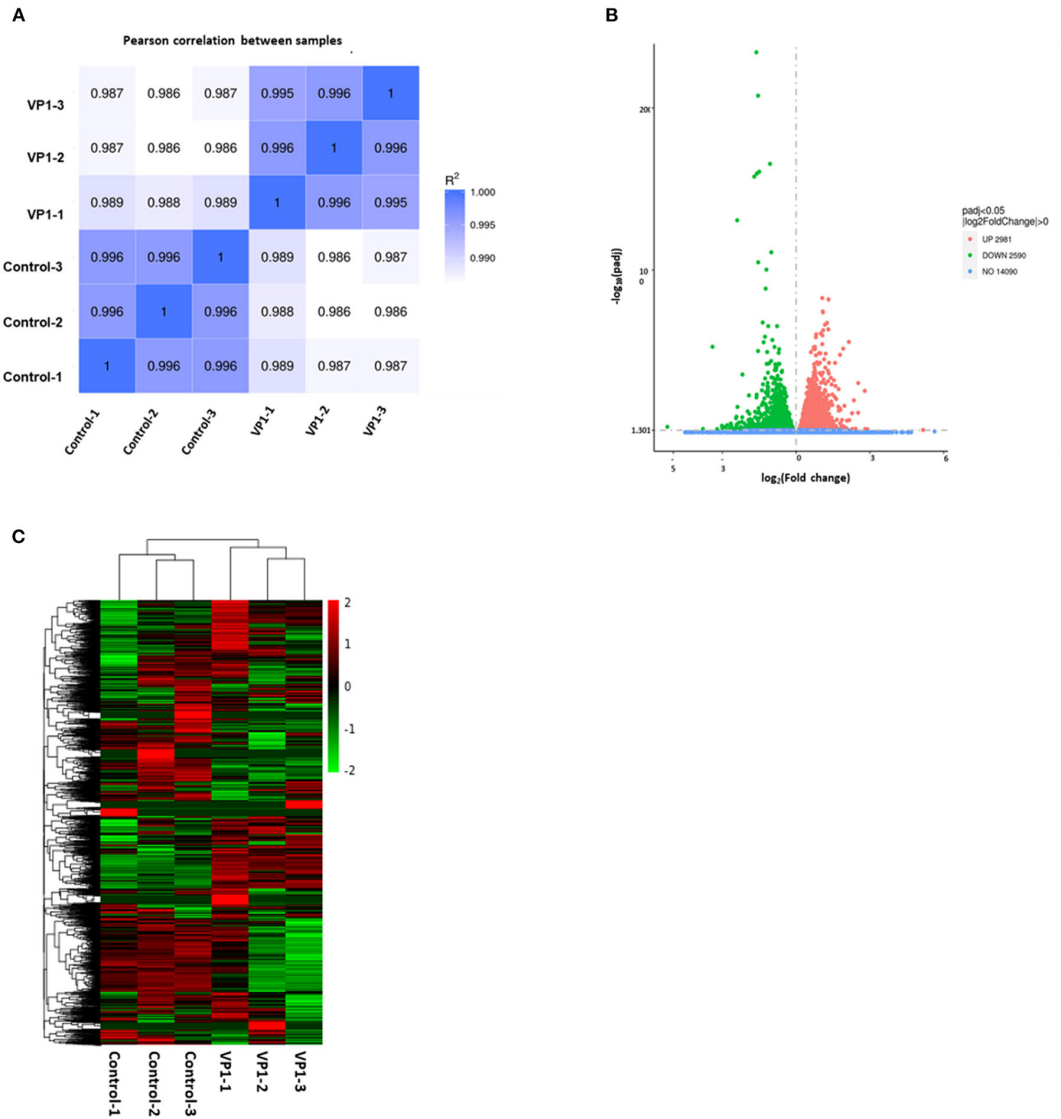
Quality control of samples and quantitative analysis of differentially expressed genes (DEGs). **(A)** Sample correlation heat map. The shades of color and numbers represent Pearson's correlation coefficient (R) values, indicating sample repeatability in the same group. **(B)** Volcano plots of the distribution of DEGs. The x-axis represents the fold change (log2Fold Change) of gene expression between the VP1 and control group, and the y-axis indicates the significance level (-log10padj) of gene expression difference. The dot diameter represents expression levels of DEGs which are either up-regulated (red) or down-regulated (green). And blue dots represent non-significantly regulated genes. **(C)** Differential gene cluster map. The x-axis in the figure is the sample name, and the y-axis is the value after the normalization of differential gene FPKM. The color scale represents gene expression abundance, with red representing more pronounced up-regulation and green representing more pronounced down-regulation.

To demonstrate that the biological experimental manipulations involved are reproducible, it was ensured that subsequent differential gene analysis yielded more reliable results. We used gene expression level correlations between samples to test the experimental reliability and the rationality of sample selection. As shown in Figure 2A, in our experiments, the R value of each sample was higher than 0.986, demonstrating high reliability. These data showed that our results are reproducible and available for subsequent analysis. As shown in Figure 2B, compared with control samples, a total of 2,981 significantly up-regulated genes and 2,590 down-regulated genes were screened under the criteria of a minimum of a *p*-value < 0.05 in the volcano plots. As shown in Figure 2C, the gene expression trends of samples from the control group and the experimental group are different, while the gene expression trends within the group are more consistent. The expression patterns of some genes are very similar and may play a role in the same cellular function. These data suggest that VP1 transfection induced dramatic gene expression changes in PK-15 cells, even in a short period.

### GO and KEGG enrichment analysis

**Figure 3:**
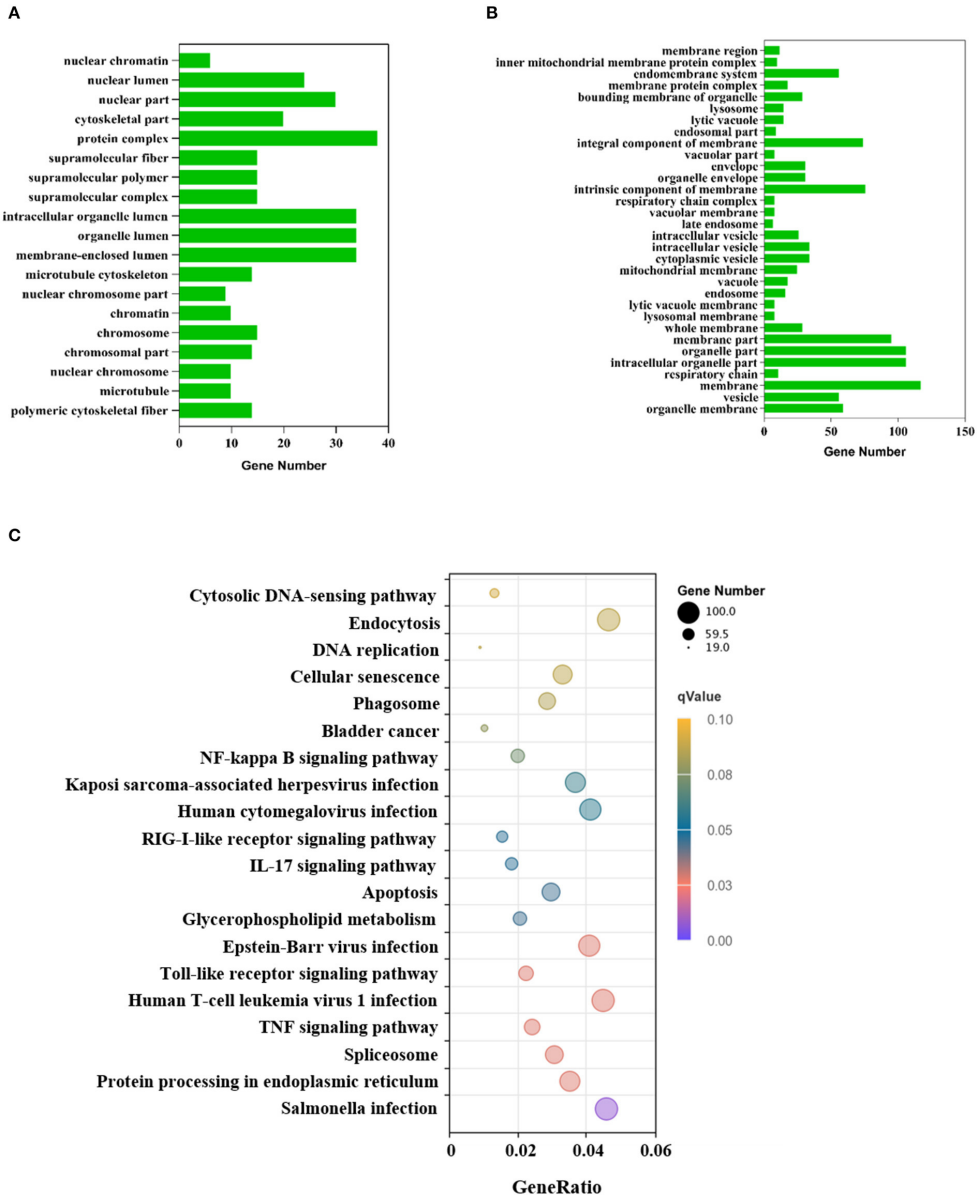
GO and KEGG annotation of differentially expressed genes. **(A)** GO functional classification of the up-regulated DEGs. The x-axis indicates the number of DEGs and the y-axis represents GO terms. From the GO enrichment analysis results, significant term plotted histograms were selected for presentation. **(B)** GO functional classification of the down-regulated DEGs. The x-axis indicates the number of DEGs and the y-axis represents GO terms. **(C)** Analysis of KEGG enrichment. The x-axis is the ratio of the number of differential genes to the total number of differential genes, the y-axis is the KEGG pathway, The dot diameter represents the number of genes annotated to the KEGG pathway, and the color represents the significant size of the enrichment.

To investigate the effect of VP1 transfection on the host's biological processes, the DEGs were used to perform GO annotation and enrichment analysis. The GO annotations of the DEGs were involved in three major categories: biological processes, cellular components, and molecular function, which were significantly enriched in 51 GO terms (*p* < 0.05; Supplementary Table S1). Up-regulated genes were significantly enriched in 19 GO terms (Figure 3A), among which protein complex (GO: 0043234), membrane-enclosed lumen (GO: 0031974) and organelle lumen (GO: 0043233) were the three GO terms with the most enriched genes. Down-regulated genes were significantly enriched in 32 GO terms (Figure 3B), among which membrane (GO: 0016020), intracellular organelle part (GO: 0044446), and organelle part (GO: 0044422) were the three GO terms with the most enriched genes. The GO results are mainly enriched in cell components, which can explain the cell structure position of the gene product when it performs its function. This indicated that VP1 could significantly affect cellular components including nuclear chromosome, microtubule and membrane structure.

KEGG enrichment analysis showed that the DEGs were mapped into 20 KEGG pathways (Figure 3C), 11 of these KEGG pathways (*p* < 0.05) were significantly enriched which were mainly related to the immune system, infectious viral disease, and signal transduction (Supplementary Table S2). The KEGG analysis showed that many genes were assigned to several pathways of host-virus interaction. In the Toll-like receptor signaling pathway, there were 48 gene expression changes. Notably, chemokine-related genes such as CCL2, CCL4, CCL5, and CXCL10 were all up-regulated, which indicated chemokines can activate cells to initiate immune responses and guide innate immune system cells. Up-regulated DEGs were mainly involved in genetic information processing (KEGG ID: ssc03040, ssc03030, ssc03013, ssc03020, ssc03410), infectious disease, environmental information processing, and cell processing (Supplementary Table S3). However, down-regulated DEGs were only involved in lipid metabolism (KEGG ID: ssc00100). These processes mainly responded to VP1 expression.

### Validation of the expression of DEGs by qRT-PCR

**Figure 4:**
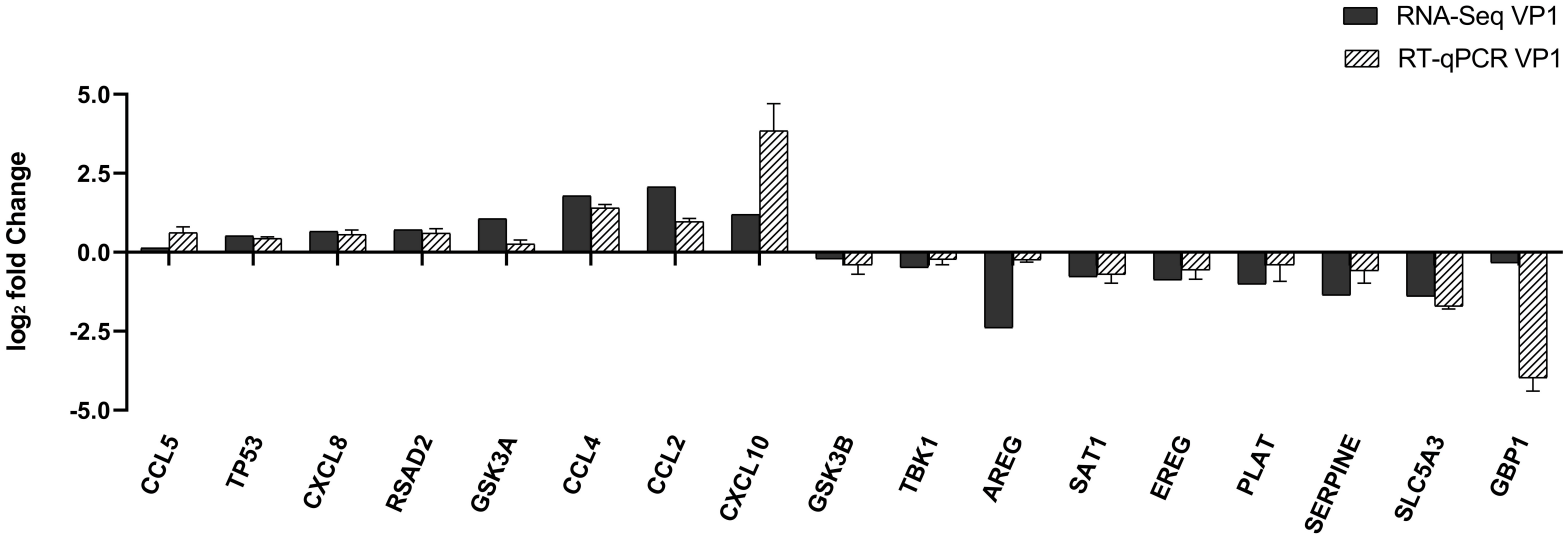
Comparison of fold changes of DEGs between RNA-Seq and qRT-PCR. PK-15 cells were transfected with pCAGGS-HA and pCAGGS-HA-VP1 for 36 h, then qRT-PCR was performed to detect the relative expression of selected DEGs. The horizontal axe represents the name and the vertical axe indicates log2-fold changes of DEGs.

To further validate the transcriptome analysis results, we performed a qPCR analysis to determine the reproducibility of the differential gene expression. GAPDH mRNA was amplified as the endogenous control. Eight up-regulated genes (CCL2, CCL4, CCL5, CXCL8, CXCL10, TP53, RSAD2, and GSK3A) and nine down-regulated genes (GSK3B, TBK1, AREG, SAT1, EREG, PLAT, SERPINE1, SLC5A3, and GBP1) were analyzed. As shown in Figure 4 and Supplementary Table S4, the qRT-PCR results corresponded with transcriptome analysis results. Interestingly, the expression of many chemokines which are important regulators of host immune responses, such as CCL2, CCL4, CCL5, CXCL8, and CXCL10 were up-regulated.

### The chemokines CCL5, CXCL8, and CXCL10 promote FMDV replication

**Figure 5:**
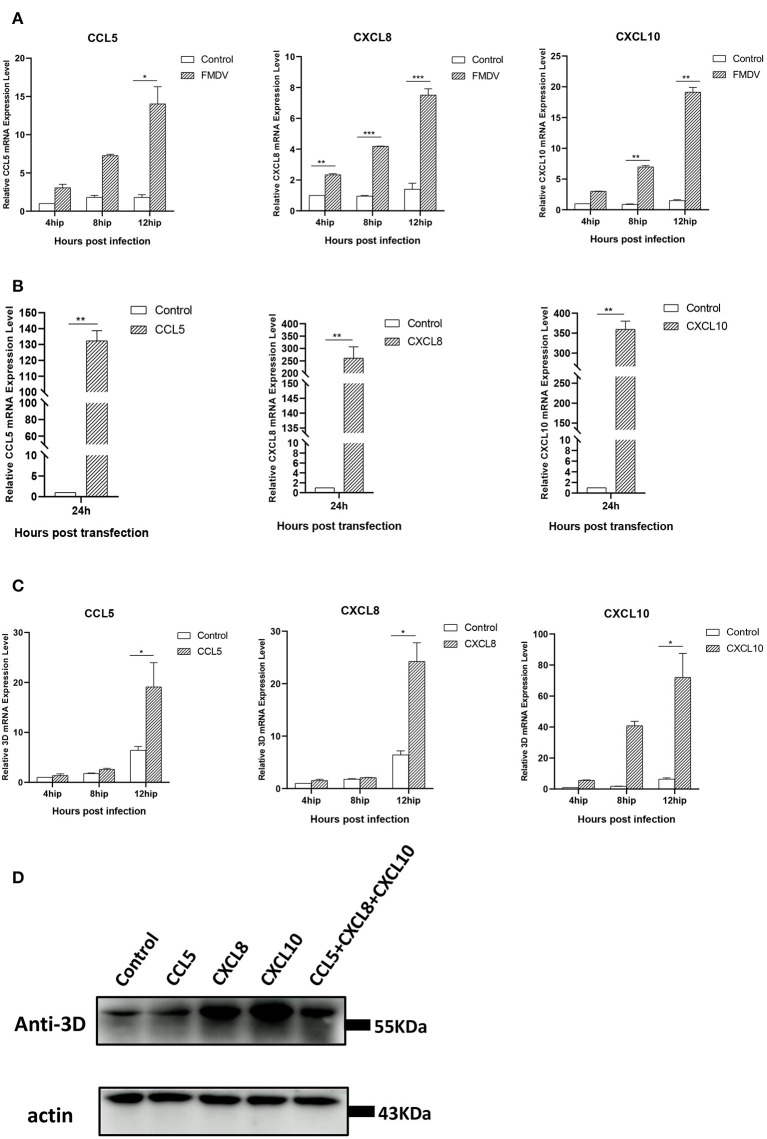
The relationship between chemokines and FMDV replication. **(A)** FMDV induces expression of chemokines in PK-15 cells. The PK-15 cells were collected at 4, 8, and 12 h after infection with FMDV at an MOI = 0.1 and then subjected to qRT-PCR assays. The mRNA levels of CCL5, CXCL8, and CXCL10 were tested. **(B)** The overexpression of chemokines in PK-15 cells. The pCAGGS-HA-CCL5/CXCL8/CXCL10 plasmids were transfected in PK-15 cells, respectively, and the expression levels of these chemokines were detected by qRT-PCR at 24 h after transfection. **(C)** Chemokines promotes FMDV replication. The pCAGGS-HA-CCL5/CXCL8/CXCL10 plasmids were transfected in PK-15 cells, respectively. At 24 h post-transfection, the cells were collected at 4, 8, and 12 h after infection with FMDV at an MOI = 0.1 and then subjected to qRT-PCR assays. The relative 3D gene mRNA levels were tested. The significant difference is marked by “*” (*P* < 0.05), “**” (*P* < 0.01), and “***” (*P* < 0.001). **(D)** PK-15 cells were transfected with pCAGGS-HA- CCL5/CXCL8/CXCL10 plasmid or co-transfected with these three plasmids for 24 h, followed by infection with FMDV for 12 h. The viral 3D protein was detected by Western blot.

Through transcriptome analysis and qRT-PCR verification, we found the expression of some chemokines was obviously increased in pCAGGS-HA-VP1 transfected PK-15 cells. Hence, we detected the mRNA levels of CCL5, CXCL8, and CXCL10 in FMDV-infected PK-15 cells at different times (4, 8, and 12 h). As Figure 5A showed, the expression of these chemokines was notably induced by FMDV and gradually increased over time of infection. The results implied that the expression of CCL5, CXCL8, and CXCL10 induced by FMDV was consistent with that induced by VP1.

Next, we constructed the eukaryotic expression plasmids of CCL5, CXCL8, and CXCL10 to explore the roles of these chemokines in FMDV infection. After transfection of these plasmids, respectively, in PK-15 cells for 24 h, the cells were followingly infected with FMDV for different times (4, 8, and 12 h). The results of qRT-PCR showed the transfection of the recombinant plasmids extremely enhanced expression levels of CCL5, CXCL8, and CXCL10 compared with control groups (Figure 5B). Accompanied by the overexpression of these chemokines, the mRNA and protein levels of FMDV 3D were obviously increased, especially at 12 h post-FMDV infection (Figures 5C,D). To verify whether there is an additive or synergistic effect of these three chemokines, we performed treatment with all 3 chemokines plasmids co-transfection to compare with single chemokine plasmid transfection. As shown in Figure 5D, the 3D protein levels of FMDV in the co-transfection group of 3 chemokine plasmids were not the highest compared with the single chemokine, indicating that there wasn't an additive or synergistic effect of these three chemokines. The above results proved the overexpression of CCL5, CXCL8, and CXCL10 promoted FMDV replication in PK-15 cells and revealed that these chemokines may play important roles in FMDV infection. Combined with the transcriptome results, we proved that FMDV upregulated the expression of these chemokines through VP1 to promote viral replication.

### Overexpression GBP1 inhibits FMDV replication

**Figure 6:**
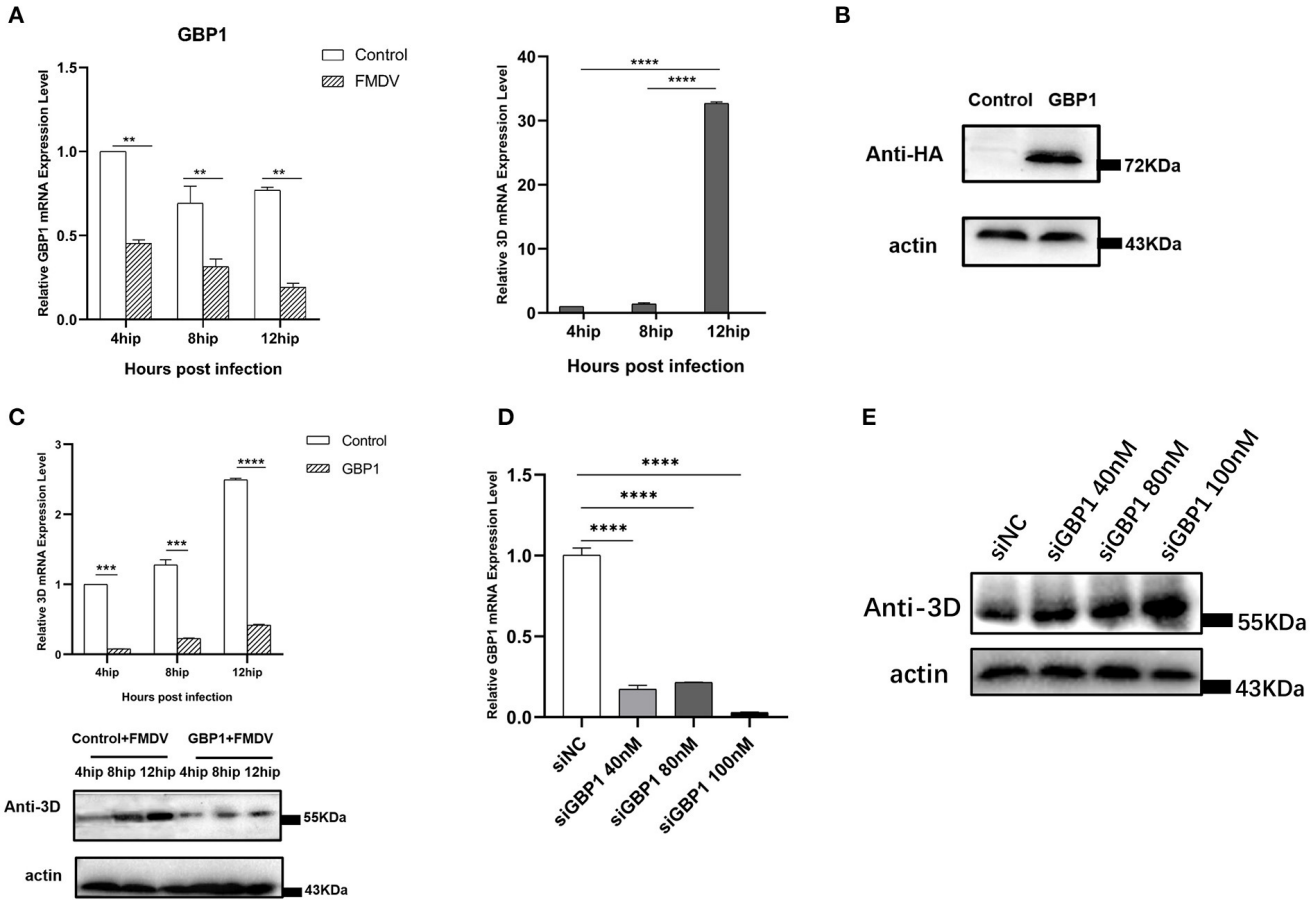
The relationship between GBP1 and viral replication. **(A)** FMDV infection downregulates GBP1 mRNA expression. After infection of PK-15 cells with FMDV for 4, 8, and 12 h, the mRNA levels of GBP1 and 3D in the samples were detected by qRT-PCR. **(B,C)** GBP1 inhibits FMDV replication. PK-15 cells were transfected with pCAGGS-HA-GBP1 plasmid for 24 h, followed by infection with FMDV for 4, 8, or 12 h. The viral RNA was determined by qPCR assay (**C**, upper panel), the GBP1 **(B)** and viral 3D protein (**C**, lower panel) was detected by Western blot. **(D,E)** Influence of GBP1 knockdown on FMDV replication. PK-15 cells were transfected with siGBP1 (siGBP1-597) in indicated concentration or siNC for 36 h, followed by infection with FMDV for 12 h. Knockdown of GBP1 mRNA was determined by qRT-PCR **(D)**, the viral 3D protein was detected by Western blot **(E)**.

In the previous transcriptome validation, we found that GBP1 was most significantly downregulated gene in the VP1 group. The GBP1, an IFN-induced GTPase, is necessary for host mediation of the immune response which exert antiviral effects against many exogenous pathogens, such as toxoplasmas, chlamydiae, bacteria, and various viruses (29). To explore the potential role of GBP1 during FMDV infection, we investigated the expression level of GBP1 in FMDV-infected cells. PK-15 cells were infected with FMDV (MOI of 0.1), and the dynamics of GBP1 were determined. The results showed that as the infection progressed, viral RNA was gradually increased, but the mRNA expression level of GBP1 was significantly decreased, confirming the negative correlation between GBP1 expression and viral replication (Figure 6A). Then, the role of GBP1 in FMDV replication was assessed. HA-GBP1-expressing plasmid was transfected in PK-15 cells for 24 h and then infected with equal amounts of FMDV (MOI of 0.1) for 4, 8, and 12 h. As the results showed, the expression level of GBP1 was fairly high (Figure 6B), and overexpression of GBP1 significantly suppressed FMDV replication in different infection times (Figure 6C). Since GBP1 overexpression suppressed FMDV replication, we examined whether interfering of endogenous GBP1 affected viral replication. The siRNA sequences targeting GBP1 were designed as described previously (29). PK-15 cells were transfected with different concentrations of GBP1 siRNA for 36 h, and then infected with FMDV (MOI of 0.1) for 12 h. The results showed that GBP1 mRNA levels were significantly reduced compare to that in the siNC-transduced control cells (Figure 6D), and knockdown of GBP1 significantly promoted FMDV replication in a dose-dependent manner (Figure 6E). In all, the consequence demonstrated that FMDV inhibited the expression of GBP1, and GBP1 could effectively constrain FMDV replication in turn. Combined with the transcriptome results, we proved that FMDV down-regulated the expression of GBP1 through VP1, thereby inhibiting the antiviral response of GBP1.

## Discussion

As a structural protein of FMDV, VP1 was located on the surface of the capsid. It could form G-H loop including a major neutralizing antigenic site and contains highly conserved RGD motifs to bind the αV family of integrin receptors. Hence, previous studies about VP1 were mainly focused on its immunogenicity for designing vaccines and its role in virus entry *via* attaching to receptors. However, the function of VP1 on host cells was rarely discussed. In this study, we found that VP1 caused 5,571 genes with significantly different expression levels by high-throughput sequencing, of which 2,981 were up-regulated and 2,590 were down-regulated (*P* < 0.05), indicating that expression of the VP1 protein caused a drastic cellular response. To identify the signaling pathways involved in the differentially expressed genes, we performed enrichment analysis by GO and KEGG databases. GO functional annotation of differential genes showed that 51 GO entries were significantly enriched after transfection, all of which involved cellular components, and 11 KEGG pathways were significantly enriched which included some innate immune pathways, apoptosis pathway, endocytosis pathway, and TNF signaling pathway (Figures 2, 3). These results proved that the VP1 proteins had an extensive influence on host cells.

The immune system, including innate and adaptive immunity, is the main protective system against the invasion, surveillance, and removal of pathogenic microorganisms. Previous studies have shown that the expression of MHC class I molecules on the cell surface is inhibited 30 min after FMDV infection, which indicates that cells infected with FMDV will immediately lose the ability to present MHC-I-related viral peptides to T lymphocytes, and this mechanism will help the virus escape from the cytotoxic immune response of the host. Besides, limiting NK cell-mediated killing is also an important mechanism for FMDV evading the cellular immune response (30). As an important component of the innate immune system, cytokines are involved in autocrine, paracrine, and endocrine signaling as immunomodulators, which are essential for the prevention of viral infections (31). As reported, the SADS CoV infection could inhibit the expression of NF-κB and create a favorable milieu for SADS-CoV replication (32). Our results showed that VP1 could trigger part of innate immune pathways such as NF-κB pathway, toll-like receptor pathway, RIG-I-like receptor pathway, and so on. The results were consistent with recent studies which found that VP1 proteins could inhibit the production of IFN-I in cells by binding to sorcin protein and TPL2 protein (19–21). In addition, VP1 protein also competitively binds to MAVS with TARF3 to inhibit the innate immune response (22). Interestingly, through qRT-PCR assays, we found that some immune-related genes such as CCL2, CCL4, CCL5, CXCL8, CXCL10, and RSAD2 were up-regulated, but several immune-related genes including GBP1 and TBK1 were down-regulated (Figure 4). The reason may be the different multiple roles of each gene in host cells and molecular mechanisms need to be further explored.

Cytokines include chemokines, interferons, interleukins, lymphokines, and tumor necrosis factors, which are produced by a variety of cells, including immune cells, such as macrophages, B lymphocytes, T lymphocytes, and mast cells, as well as other cells (33). In a previous study, sequencing of the transcriptome at the early stage of FMDV infection revealed that the chemokines TNF, CXC chemokine (CXCL2), CC chemokine (CCL20, CCL4), interleukin (IL6), and NF-κB inhibitor α (NFKBIA), were significantly up-regulated shortly after FMDV infection (34). In our study, chemokines, including CXC chemokines (CXCL8, CXCL10), and CC chemokines (CCL2, CCL4, CCL5), were up-regulated after transfection with VP1 plasmid (Figure 4), and CCL5, CXCL8, and CXCL10 were also up-regulated after FMDV infection (Figure 5A), which indicated that VP1 play a role in the process of FMDV up-regulated chemokines. Besides, overexpression of CCL5, CXCL8, and CXCL10 enhanced FMDV infection (Figure 5C). For different viruses, these factors play various roles. The studies showed that CCL5 could inhibit Influenza A and HCV infection (35, 36). In contrast, CCL5 is required for ZIKV to persistently infect human brain Ecs (37). CXCL8 could mediate productive infection of HIV-1 in cells *via* receptors CXCR1 and CXCR2 and also could be induced by Porcine epidemic diarrhea virus nsp4 which in turn inhibited virus infection (38, 39). Furthermore, CXCL10 was also reported that it could inhibit viral replication through the recruitment of natural killer cells in coxsackievirus B3-induced myocarditis and CXCL10/IP-10 inhibited dengue virus replication *via* competing with dengue virus for binding to heparan sulfate on the cell surface (40, 41). Therefore, discovering the influences and mechanism of CCL5, CXCL8, and CXCL10 on FMDV infection would be the key point in our further study.

The replication cycle of FMDV in its host is very short, and its virulence factors alter the host cells environment to promote viral replication by inactivating host factors and blocking their function (42). Many FMDV viral proteins have been shown to play a suppressive role in the host immune response, thereby ensuring viral replication in the host (43, 44). These virulence factors inhibit the host antiviral immune response in a variety of ways, mainly including: inhibiting the synthesis of host proteins, blocking the synthesis of interferons, reducing the expression of interferon-stimulated genes (ISGs) and pro-inflammatory cytokines. For example, overexpression of VP1 inhibits type I IFN production and response *via* interacting with host proteins (19, 22, 45); hepatitis C virus obviously suppresses the expression of ISG such as GBP1 which in turn interferes with virus infection by interacting with the viral protein NS5B (46). Our results also showed that VP1 protein obviously down-regulated GBP1 expression (Figure 4) and the same consequence was obtained under FMDV infection (Figure 6A). We speculate that the mechanism of VP1 down-regulating the transcription level of GBP1 may be that VP1 affects the promoter activity of GBP1 or affects the stability of GBP1 mRNA. At the same time, we found that overexpression of GBP1 significantly inhibited the replication of FMDV at different infection time points (Figure 6C). It could be speculated that FMDV altered the host cells environment to promote viral replication by down-regulating the expression of host protein GBP1 and blocking its function. But the detailed mechanism needs further exploration.

Our results provide a comprehensive overview of the host response to VP1 protein of FMDV, presenting significant insights into the interactions between VP1 and host cells. Furthermore, the transcriptome analyses broaden our understanding of FMDV pathogenesis and screening for genes such as GBP1 that confer resistance to FMDV infection.

## Data availability statement

The datasets presented in this study can be found in online repositories. The names of the repository/repositories and accession number(s) can be found in the article/Supplementary Material.

## Author contributions

LY and XX designed the experiments and wrote the manuscript. LY, LL, JS, HC, and TF carried out the experiments. LY analyzed the data analysis. XX, YL, and LK performed writing-review and editing. CS checked the manuscript. All authors read and approved the final.

## Funding

This work was supported by Research Grants from National Natural Science Foundation of China (Grants 31460667 and 31860038) and Jiangxi Province (Grants 20192BAB214001, 20161BBF60084, and GJJ180240).

## Conflict of interest

The authors declare that the research was conducted in the absence of any commercial or financial relationships that could be construed as a potential conflict of interest.

## Publisher's note

All claims expressed in this article are solely those of the authors and do not necessarily represent those of their affiliated organizations, or those of the publisher, the editors and the reviewers. Any product that may be evaluated in this article, or claim that may be made by its manufacturer, is not guaranteed or endorsed by the publisher.
