# Supplementary material for: Foot-and-mouth disease virus VP1 promotes viral replication by regulating the expression of chemokines and GBP1
Source: Front Vet Sci. 2022 Jul 22;9:937409. doi: 10.3389/fvets.2022.937409 (PMC9353127; doi:10.3389/fvets.2022.937409)
Supplement: Supplementary file 5 [file Table_5.DOCX]

***Supplementary Material***

1. **Supplementary Tables**

**Supplementary Table S1**. GO functional significant enrichment

**Supplementary Table S2**. 20 KEGG pathway enrichment of differentially expressed genes

**Supplementary Table S3.** KEGG functional categorization of up-regulated DEGs

**Supplementary Table S4.** Comparison of fold changes of DEGs between VP1 transfected cells and negative controls by qRT-PCR and RNA-Seq
